# Apps and eating disorders: A systematic clinical appraisal

**DOI:** 10.1002/eat.22398

**Published:** 2015-02-27

**Authors:** Christopher G. Fairburn, Emily R. Rothwell

**Affiliations:** ^1^ University of OxfordOxfordUnited Kingdom

**Keywords:** Apps, mobile technology, cognitive behavior therapy, assessment, self‐monitoring, recording, treatment, smartphones, eating disorders

## Abstract

**Objective:**

Smartphone applications (apps) are proliferating and health‐related apps are particularly popular. The aim of this study was to identify, characterize, and evaluate the clinical utility of apps designed either for people with eating disorders or for eating disorder professionals.

**Method:**

A search of the major app stores identified 805 potentially relevant apps, of which 39 were primarily designed for people with eating disorders and five for professionals.

**Results:**

The apps for people with eating disorders had four main functions. Most common was the provision of advice, the quality of which ranged from sound to potentially harmful. Five apps included self‐assessment tools but only two used methods that would generally be viewed as reliable. Four apps had the self‐monitoring of eating habits as a major feature. Entering information into these apps could be accomplished with varying degrees of ease, but viewing it was more difficult. One app allowed the transfer of information between patients and clinicians.

**Discussion:**

The enthusiasm for apps outstrips the evidence supporting their use. Given their popularity, it is suggested that clinicians evaluate app use as part of routine assessment. The clinical utility of the existing apps is not clear. Some are capable of tracking key features over time, but none has the functions required for analytic self‐monitoring as in cognitive behavioral treatments. The full potential of apps has yet to be realized. Specialized apps could be designed to augment various forms of treatment, and there is the possibility that they could deliver an entire personalized intervention. © 2015 The Authors. International Journal of Eating Disorders published by Wiley Periodicals, Inc. (Int J Eat Disord 2015; 48:1038–1046)

## Introduction

“Apps” (shorthand for applications) are specialized self‐contained software programs designed for use on smartphones and other mobile devices such as tablet computers. Despite having only been in existence since 2008, over three million apps are available^1^ and this figure is rapidly increasing.^2^ Apps have been designed for many different purposes, including health care, with almost one fifth of smartphone users thought to have at least one health‐related app on their phone.^3^ This figure is expected to reach 50% by 2018.^4^ Nevertheless, many health‐related apps are barely used: indeed, over fifty percent are downloaded less than 500 times.^5^

The most common type of problem addressed by health apps are “mental health and behavioral disorders.”^5^ Many difficulties are addressed including anxiety disorders, depression, and excessive alcohol use, and the apps have a variety of functions including supplying information, self‐assessment, self‐monitoring, and the provision of advice or treatment.^6^

The present study had three aims. The first was to identify all available apps (in English) that are primarily designed for people with eating disorders or for professionals helping people with eating disorders. The second aim was to assess their popularity and to characterize and evaluate them on the basis of the functions that they claimed to serve. The third aim was to conduct a systematic appraisal of the clinical utility of the apps including those designed for use by clinicians.

## Methods

### Identification of the Apps

A two‐step process was used. First, all potentially relevant apps were identified, as of July 31, 2014, by entering the terms “eating disorder,” “eating disorders,” “anorexia,” “anorexia nervosa,” “bulimia,” “bulimia nervosa,” “binge,” and “binge eating” into the search boxes of the official application stores of the five major smartphones: iPhone, Android, BlackBerry, Nokia, and Windows Mobile. In addition, the Amazon App store was searched as it is another major source of Android apps. A supplementary search was conducted by entering “Apps for eating disorders” into the search engine Google and identifying the apps selected by websites claiming to list apps for eating disorders.

The second step involved the two authors independently reviewing each of the English‐language apps identified in the first step to select those primarily designed for people with eating disorders or for professionals helping those with eating disorders. Where there were inconsistencies, these were resolved by discussion.

### Assessment of the Popularity of the Eating Disorder Apps

This was estimated using data from Xyologic, a mobile app search engine that provides app download data. If an app was not listed on Xyologic or it was reported as “<1,000,” download data from Google Play was used. If an app was downloadable from more than one application store, the total sum of downloads was calculated.

### Characterization of the Functions of the Eating Disorder Apps

Each of the apps identified in the second step was assessed. Their functions were grouped into four categories: (i) Provision of information; (ii) Self‐assessment; (iii) Self‐monitoring; and (iv) Provision of advice or treatment.

### Evaluation of the Leading Apps

#### Provision of Information

Both authors independently evaluated the quality of the information furnished. It was categorized as “Good” if it was consistent with that provided by reliable sources such as the American Psychiatric Association's DSM‐5^7^; “Variable” if there were some errors or significant omissions; and “Poor” if the information was positively misleading or frankly wrong. Discrepancies between the authors were resolved by discussion.

#### Self‐Assessment

This was tested by the second author. This involved her answering the assessment questions as if she had anorexia nervosa, and then repeating the process as someone with bulimia nervosa, and finally as someone with a mixed state with features of both eating disorders. The questions were also answered as if no significant eating disorder psychopathology was present. The apps were judged as providing a “Good” assessment if the questions asked and responses supplied were appropriate. They were classed as “Variable” if there were some errors or significant omissions, and “Poor” if either the questions were inappropriate or the output was misleading or wrong.

#### Self‐Monitoring

Self‐monitoring has two main purposes. The first is the ongoing monitoring of key clinical features—in the case of eating disorders, eating habits such as binge eating and purging—to track their presence and severity, and whether they are changing over time (“psychopathology tracking”). This requires that the relevant information is readily entered, and subsequently reported in a form that is easy to access and understand. The second use is to help patients change. Cognitive behavioral treatments (CBT) in particular use the real‐time self‐monitoring of eating habits to help patients gain a better understanding of their eating problem and what influences it (“psychopathology analysis”) and to help them intervene in the moment.^8^, ^9^ This form of monitoring therefore requires that the relevant information can be easily entered in real time (especially eating habits and the context in which they occur), and a form of reporting that allows the user to look back repeatedly at what has happened so far in the day and on previous days.

The self‐monitoring apps were tested with these two purposes in mind. Anonymized data from the CBT records of three patients were entered; one with anorexia nervosa, the second with bulimia nervosa, and the third with a mixed state. This involved the second author taking the role of each of these patients in turn and inputting in real time their monitoring record data. The ease of entering information was assessed as was the type of output generated.

#### Provision of Advice or Treatment

The advice proffered by the apps was evaluated from two perspectives. The first author took the perspective of an expert clinician whereas the second took the perspective of someone with an eating disorder. The quality of the advice was judged from both perspectives and was rated using the same categorical scheme as above.

## Results

### Identification of the Eating Disorder Apps

Eight hundred and five apps were identified in the first step, the main source being the Google Play store. The supplementary internet search resulted in the identification of three additional apps, none of which was available to download. **Table** 1 shows the results of the second step. The two authors judged 39 of the apps (4.84%) to be primarily designed for those with an eating disorder and a further five (0.62%) to be intended for professionals. The remaining 761 apps had a wide variety of primary functions including helping control excessive drinking; providing dietary advice or help with exam revision or weight loss; training in cooking, hypnosis or relaxation; and assistance in identifying restaurants.

**Table 1:** Eating disorder apps and their source

	Apps Designed Primarily for People With an Eating Disorder	Apps Designed for Professionals	Excluded Apps^a^
Amazon	10	1	28
Blackberry	3	0	140
GooglePlay	32^b^	3	557
iTunes	47	5	30
Nokia	13	0	13
Windows	30	0	26
Total^c^	39	5	781

a Including duplicates across stores.

b One app was excluded as it failed to download.

c Each app is counted only once (i.e., duplicates have been excluded).

### Popularity of the Apps

Download data could be obtained on 41 of the 44 identified apps. The apps differed greatly in their number of downloads (see **Table** 2). Thirty‐three (80.5%) had been barely used (≤5,000 downloads) whereas two (4.9%) had been downloaded over 50,000 times.

**Table 2:** Apps designed primarily for people with eating disorders (as of 31 July, 2014)

App	Target User Group	Downloads (*N*)	User Rating (1–5)^d^	Number of User Ratings (*N*)^d^	Functions				
					Provision of Information	Self‐Assessment	Self‐Monitoring		Provision of Advice or Treatment
							Tracking	Analysis	
*Anorexia Books* e	AN	1,000–5,000	2.5	4					
*Anorexia Study*	AN	2,700	4.5	5	b				
*Anorexia Tips, Free Dev*.^f^	AN	NA	2.5	4					a
*Anorexia Tips, InfoApps247*	AN	1,200	3.5	8	a				
*Anorexia Tips, Minh T Trans*	AN	3,300	2.6	23					a
*Anorexia Tips, The Best of Best Apps* f	AN	5,000	3.5	16					a
*Anorexia Understood*	AN	NA	1	1	b				
*Anorexia, Bulimia, Binge Eating Test*	EDs	2,900	3.5	5	b	c			c
*Appetite Antidote*	BED and BN	<1000	4	1					a
*Before I Eat*	BED and BN	5,200	4.2	35					a
*Binge Eating and How to Stop*	BED and BN	<1,000	3.2	4					b
*Binge Eating Disorder, InfoApps247*	BED	<1,000	4	1	b				a
*Binge Eating Disorder, Power Apps LLC*	BED	<1,000	NA	NA	a				c
*Binge Eating Help*	BED	NA	NA	NA	b				b
*BingeBoard* e	BED and BN	1–5	NA	NA					
*Breaking Bulimia*	BN	<1,000	2	1	c				c
*Bulimia Help*	BN	100–500	5	1	a				
*Cbutterfly Forum* e	EDs	1,300	3	10					
*Daytime affimations for overcoming binge eating*	BED	1,500	NA	NA					a
*Discovery in recovery*	EDs	4,700	NA	NA					a
*Do I have an Eating Disorder?*	EDs	3,900	NA	NA		b			
*Eating D*	EDs	<1,000	3.6	5					b
*Eating Disorder Assessments*	EDs	<1,000	4.2	4		b			c
*Eating Disorder Current Concept*	EDs	2,900	NA	NA	b				
*Eating Disorder Test*	EDs	10–50	NA	NA		a			
*EATINg DISORDEr TESt (quick)*	EDs	<1,000	NA	NA		c			
*Eating Disorders, Joshua Steinberg*	Clinicians	15,000							
*Eating Disorders, Master and Bull Digital Arts*	Teens with EDs	<1,000	NA	NA	b				b
*Eating Recovery Center Events*	Clinicians	1,200							
*Emotes for Disordered Eating*	EDs	1,300	4.3	6			c	b	
*FIND HELP 4 EATING DISORDERS* e	EDs	1,000	2.9	7					
*iCounselor: Eating Disorder*	EDs	7,100	3	36					b
*Journal of Eating Disorders*	Clinicians	2,700							
*Kissy Project*	EDs	11,000	4.2	5					a
*Males with Eating Disorders*	Clinicians	<1,000							
*MealJournal*	EDs	1,500	NA	NA			b	a	
*Overcoming Eating Disorders*	EDs	<1,000	NA	NA	c				c
*Overeating cure‐ battle against bulge eating*	BED	<1,000	NA	NA	b				c
*Recovery Record*	EDs	361,000	5	1,956			c	b	c
*Recovery Record Clinician*	Clinicians	5,000							
*Rise Up + Recover*	EDs	66,000	5	173			b	b	a
*Stop Binge Eating: Lose Weight*	BED	7,000	3	30					a
*Thinspo* e, f	EDs	19,000	4.3	609					
*Ultimate weight loss hypnosis*	EDs	<1,000	1	2					a

Notes: AN, anorexia nervosa; BED, binge eating disorder; BN, bulimia nervosa; EDs, all eating disorders; NA, Not available.

a Poor quality overall.

b Moderate quality overall.

c Good quality overall.

d If there was a rating on more than one app store, the rating from the store with the greatest number of ratings was used. A rating of 1 is low and a rating of 5 is high.

e Apps with no entries had functions other than those listed in the table.

f Pro‐anorexia apps.

### Functions

#### Provision of Information

Of the 39 apps for people with eating disorders, 13 (33.3%) provided information (see **Table** 2). The quality and quantity of this information varied greatly. Two of these apps (15.4%) were judged to provide good information, in eight (61.5%) it was judged to be variable, and in three (23.1%) it was viewed as positively misleading. For example, one app described anorexia nervosa as a product of “brain washing” (*Anorexia Tips; InfoApps247*) and another stated that binge eating primarily occurs in men (*Binge Eating Disorder; Power Apps LLC*)

#### Self‐Assessment

Five apps (12.8%) had this function. Two (40.0%) were judged to provide a good assessment, whereas in the other three (60.0%) it was rated as variable or poor.

#### Self‐Monitoring

Four apps (10.3%) allowed users to monitor their eating habits. These apps were assessed in detail (see below).

#### Provision of Advice or Treatment

Twenty‐four of the apps provided advice (61.5%). Seven (29.2%) were judged to provide good advice; in five (20.8%) the advice was variable; and in 12 (50.0%) the advice was poor and in some instances potentially harmful. For example, *Anorexia Tips (Free Dev.)*, in a section for people with anorexia nervosa states: “Make yourself lunch. A big nice sandwich with juice and pack of chips. Then when you get to school, give it away to someone who forgot theirs.” Three apps (12.5%) provided advice that was tailored to information provided by the user.

#### Additional Functions

Five of the 39 apps (12.8%) did not serve any of these functions but had other functions instead. These included being supplied with daily images of “real girls”; allowing users to write to other people with eating disorders; and providing information about nearby sources of treatment.

### Apps for Self‐Monitoring

#### Emotes for Disordered Eating

This app is a “Self‐monitoring log for those who have been or are currently undergoing treatment for eating disorders.” It allows users to monitor their eating habits, as well as relevant urges. Additional information can also be recorded, and entries can be emailed to another person (see **Table** 2).

The app was found to be easy to use. It was similar to the monitoring sheets used in CBT in terms of the information that could be entered. The app generated a summary graph of various behavior that provided a clear visual representation of their frequency and any changes over time. In contrast, viewing eating habits earlier in the day was difficult as each entry had to be repeatedly clicked to see what was eaten and the context in which it occurred (as otherwise only the date and time were visible).

#### MealJournal

This app is “Designed to help those overcoming eating disorders.” Meals and thoughts can be recorded using a photo, a 10 s audio clip, a video, or as text. Entries can be tagged as binge, purge, or exercise events, and past meals and “events” can be reviewed. Text entries can be emailed to any email address.

The app was quicker to fill in than a CBT monitoring sheet as less information was sought and there was the option to take a photograph of the food to be consumed. There was no way to note the context or any accompanying thoughts or feelings, nor was it possible to chart changes over time or eating habits earlier in the day.

#### Recovery Record

This app states that it is “The smart companion for managing your journey to recovery for eating disorders .....” It allows users to record meals and snacks, thoughts and feelings, and a meal plan.

The app was found to be laborious to complete due to the extensive number of questions routinely asked. It took longer to enter details of a meal than it would using a CBT monitoring sheet. Recording the exact time of eating was not possible, thereby precluding fine‐grain recording. Some questions had default answers programmed which made it easy to inadvertently provide false information. A quick option for entering a meal or snack was available but this prevented the recording of many other features. A mandatory aspect of the app was the recording of the “adequacy” of what is eaten. The meaning of this term was not explained. There was the option of receiving prompts to record meals. In common with *Emotes for Disordered Eating*, the frequency of certain behavior could be tracked in the form of charts although these were difficult to interpret. Looking back at eating habits earlier in the day and what might have influenced them was also difficult because of the number of clicks required and, in common with all apps, the small screen size. A way round this problem was for users to access their data from the *Recovery Record* website as it was presented there more clearly. A distinctive feature of the app was the fact that information could be exchanged two‐way between the app and a linked treatment team.

#### Rise Up + Recover: An Eating Disorder Monitoring and Management Tool for Anorexia, Bulimia, Binge Eating, and EDNOS

This app states that it is “Just what you need on your journey to recovery.”

The app was easy to use and flexible in terms of what information could be entered. It had a prompt function similar to *Recovery Record*. Tables summarizing the user's data could be exported as PDFs. In common with the other apps, it was difficult viewing eating habits earlier in the day due to the number of clicks required.

### Apps Designed for Clinicians

#### Eating Disorders, Joshua Steinberg

This app provided an outline of eating disorders as classified in DSM‐IV. It advocated screening using the SCOFF questions, and provided limited information about assessment and medical evaluation, together with guidance regarding whether treatment should be as an inpatient or outpatient. It was judged to be of value to non‐specialist clinicians, but in need of updating and refining.

#### Eating Recovery Center Events

This app listed events sponsored by the “Eating Recovery Centre.”

#### Journal of Eating Disorders

This app listed articles published in the *Journal of Eating Disorders*. These could be downloaded as PDFs.

#### Males with Eating Disorders

This app comprised a brief single‐author eBook on the topic of males with eating disorders.

#### Recovery Record Clinician

This is the clinician counterpart to the *Recovery Record* app. It can be linked with the Recovery Record apps of patients allowing the two‐way transfer of information and messages. Inspecting patients' summary charts was straightforward but viewing their eating habits within the day and from day‐to‐day was difficult due to the number of clicks required.

## Discussion

Although apps are a recent invention, there are a huge number of them and some are heavily used. The purpose of this study was to identify and characterize the apps designed primarily for people with eating disorders or eating disorder professionals. We also assessed their clinical utility.

We identified over 800 apps (in English) on entering eating disorder search terms into the search boxes of the major app stores. This vast number of potentially relevant apps is likely to confuse, if not overwhelm, those searching for the first time. On examination of the apps just 39 had been primarily designed for people with eating disorders and of them the majority were little used with just eight having been downloaded 5,000 or more times. An additional five apps were designed for eating disorder professionals. These were heterogeneous in nature.

The 39 apps for people with eating disorders had four major functions, the most common being the provision of advice. Often the advice was less than satisfactory and in some instances it was potentially harmful. Next most common was the provision of information and this varied greatly in quality. Few apps were judged to provide sound information. Five apps allowed users to assess the presence and severity of any eating disorder psychopathology, but only two used methods that would generally be viewed as reliable.

Self‐monitoring is likely to be the function of most interest to clinicians. As noted earlier, this has two main uses, psychopathology tracking and psychopathology analysis. Two questions therefore arise. Could the conventional method of self‐monitoring using written records or questionnaires be replaced by app‐based recording, and if so does this apply equally to psychopathology tracking and psychopathology analysis? App‐based recording has potential advantages as most patients have smartphones and they use them frequently. It might, therefore, be simpler for them to record using a smartphone app than using written records and making entries might be accomplished more easily, accurately, and discretely.

We assessed the four self‐monitoring apps from two perspectives, that of the user or patient and that of a clinician or therapist, and with both psychopathology tracking and psychopathology analysis in mind. The user experience was not as positive as we had expected. While it was convenient not having to carry a monitoring record, recording using an app was in most instances no faster and it was frustrating in many ways. The apps were largely inflexible in the way that information had to be entered and pre‐set options sometimes prevented an accurate description of associated thoughts, feelings, and circumstances. Three apps provided summaries of the information entered thereby permitting psychopathology tracking, but none was suited to psychopathology analysis. Looking back at what had happened earlier in the day was laborious due to the number of clicks required and seeing the output was difficult because of the small screen. *Recovery Record* addressed this problem by giving users access to the *Recovery Record* website on which their data could be viewed with fewer clicks. The problem of screen size remained, however, unless equipment with a larger screen was employed but then the convenience of solely using a phone was lost. Neither of these difficulties affects those who use written monitoring records as they are always easy to access and view.

The relative pros and cons of written versus app‐based recording therefore depends largely upon its purpose. If the goal is psychopathology tracking or in recording solely what the person is eating (i.e., the goal is to obtain “food records”), then app‐based recording has some advantages over written recording. The converse applies to psychopathology analysis.

It is important not to forget that the great majority of app users are recording of their own volition and not in the context of treatment. Is this helpful, harmless, or is there a reason to be concerned about it? An inordinate interest in eating and related phenomena is well‐known to be characteristic of people with eating disorders, and often this extends to keeping detailed records of food intake, exercising, body weight, and other phenomena. Apps provide sufferers with a new means of doing this. This is probably harmless unless it encourages eating disorder behavior or it delays people seeking help. The exchange of information with other sufferers, a property of several apps, is not necessarily benign as another well‐known characteristic of people with eating disorders is competitiveness and in particular competing in attempts to diet, exercise, and lose weight. Putting users in touch with each other might intensify such behavior and it might also result in the acquisition of new forms of psychopathology.

The ability to exchange information between a patient's app and that of their clinician is another function that might interest clinicians. This property is peculiar to *Recovery Record*. It allows the ongoing tracking of patients' psychopathology and it is accompanied by the ability to exchange messages in real time. This has possible drawbacks. We suspect that few clinicians will want to hear from their patients 24 h a day, and is this degree of accessibility in the patient's interest or might it encourage dependence?

There has been one other appraisal of eating disorder apps and its aims complemented those of the present study. Juarascio et al.^10^ used a similar search strategy to detect “treatment‐focused” eating disorder apps. Six were identified and each was assessed from two perspectives; its use of procedures taken from evidence‐based treatments and the extent to which it capitalized on advances in smartphone technology. It was found that a wide range of treatment procedures were delivered by the six apps, some derived from evidence‐based interventions (particularly CBT) and others not, but the interventions were perfunctory in nature comprising a few sentences of standard text. Even if they had been more sophisticated in form, their likely effectiveness may be questioned as the evidence‐based psychological treatments from which they were drawn are not a mere hugger‐mugger of techniques; rather, the procedures are designed to be personalized and used sequentially in a systematic way. As regards the apps' use of modern smartphone technology, Juarascio et al. concluded that their functionality was “very limited,” a view that we share.

The present study has certain strengths and weaknesses. Its strengths include the systematic search for all available apps and their evaluation from both the user and clinician perspective. Another strength is the appraisal of the clinical utility of the apps. The main weakness is the fact that the functional testing was limited with respect to the amount of data entered and the fact that the judgments were based on the views of just one clinician and one sufferer/patient. Nevertheless, a number of conclusions appear warranted.

Starting from the perspective of clinical practice, it is suggested that as part of routine assessment, clinicians should ask patients about their use of apps as they may have obtained unreliable information, feedback, or advice, and they may be engaged in unhelpful forms of communication with other sufferers. Clinicians may want to consider using an app in their management of patients as certain apps are capable of tracking changes in psychopathology (e.g., changes in binge eating, self‐induced vomiting, etc) but it must not be forgotten that recording in this way is demanding of the patient and not necessarily benign. Unfortunately, none of the existing apps is capable of psychopathology analysis so CBT therapists in particular will find that apps are a poor substitute for the conventional written record. In this specific context, the option of app‐based recording seems to be a solution in search of a problem as, in our experience, compliance with written monitoring is high if the therapist is implementing CBT well.^8^

The second set of conclusions concerns researchers. Apps need investigating. Apps provide a new means of assessing psychopathology, either on an ongoing basis or at intervals. The validity and clinical utility of these assessments need to be determined. Similarly, there have been no published studies of the therapeutic effects of any of these apps^11^ and, indeed, very few of mental health apps in general.^12^ This needs to be rectified. Apps could be used in treatment in various ways; for example, they could augment face‐to‐face treatments by making them more effective or efficient, or they could possibly deliver an entire personalized (tailored) intervention. This latter possibility is especially interesting as it would have enormous advantages in terms of scalability and access.

The final conclusion concerns organizations serving those with eating disorders. A useful additional service that they could provide would be to maintain an up‐to‐date list of the leading eating disorder apps in which their strengths, weaknesses, and potential risks are specified. This would be of great value to users and clinicians alike.
